# Editorial: TMS application in both health and disease

**DOI:** 10.3389/fnhum.2022.1110274

**Published:** 2023-01-04

**Authors:** Ana Dionísio, Sandra Carvalho, Joao Castelhano

**Affiliations:** ^1^Coimbra Institute for Biomedical Imaging and Translational Research, University of Coimbra, Pólo das Ciências da Saúde, Coimbra, Portugal; ^2^Institute for Nuclear Sciences Applied to Health, Pólo das Ciências da Saúde, University of Coimbra, Coimbra, Portugal; ^3^Translational Neuropsychology Laboratory, Department of Education and Psychology, William James Center for Research, University of Aveiro, Campus Universitário de Santiago, Aveiro, Portugal

**Keywords:** brain stimulation, therapeutic applications, medical imaging, transcranial magnetic stimulation (TMS), neuronal mechanism

Transcranial magnetic stimulation (TMS) can be useful for therapeutic purposes for a variety of clinical conditions. Numerous studies have indicated the potential of this non-invasive brain stimulation technique to recover brain function and to study physiological mechanisms. Following this line, the articles contemplated in this Research Topic show that this field of knowledge is rapidly expanding and considerable advances have been made in the last few years. There are clinical protocols already approved for Depression (and anxiety comorbid with major depressive disorder), Obsessive compulsive Disorder (OCD), migraine headache with aura, and smoking cessation treatment but many studies are concentrating their efforts on extending its application to other diseases, e.g., as a treatment adjuvant. In this Research Topic we have the example of using TMS for pain, post-stroke depression, or smoking cessation, but other diseases/injuries of the central nervous system need attention (e.g., tinnitus or the surprising epilepsy). Further, the potential of TMS in health is being explored, in particular regarding memory enhancement or the mapping of motor control regions, which might also have implications for several diseases.

TMS is a non-invasive brain stimulation technique that can be used for modulating brain activation or to study connectivity between brain regions. It has proven efficacy against neurological and neuropsychiatric illnesses but the response to this stimulation is still highly variable. Research works devoted to studying the response variability to TMS, as well as large-scale studies demonstrating its efficacy in different sub-populations, are therefore of utmost importance. In this editorial, we summarize the main findings and viewpoints detailed within each of the 12 contributing articles using TMS for health and/or disease applications.

The developments in the last two decades have been impressive and as has been shown by Hua et al. a repetitive TMS protocol can have the potential to improve associative memory in health, which can be relevant for many neurological conditions.

In the line of using brain stimulation in healthy volunteers, Ngetich et al., provided evidence for the use of intermittent theta-burst stimulation (iTBS) in visual-spatial working memory enhancement, as well as the safety and effective profile of this technique for investigating the causal role of particular brain areas in specific psychological processes.

Brain stimulation can in fact be used to investigate the neural basis of distinct tasks. Yue and Martin, examined the effects of TMS in working memory (WM) and showed an effect of TMS on task performance, specifically on response time, in a phonological WM recognition task. With their work, they pointed out the relevance of assessing if the memory representations are restricted to local areas or distributed in a network, through studies of functional connectivity or multi-focal brain stimulation (Yue and Martin).

On the other hand, there is the use of TMS in disease, to ameliorate symptoms of e.g., post-stroke depression (PSD). PSD constitutes an important topic of research, given the major prevalence of stroke and its detrimental impact on quality-of-life. Thus, Zhu et al. explored the effects of TMS as an adjuvant to citalopram for post-stroke depression treatment. They found that, TMS in combination with citalopram were able to ameliorate symptoms of depression, as well as the neuropsychological function, in people with PSD. However, the outcome measures or evidence of treatment response of different protocols are still lacking. Strafella et al. provided a systematic review on using EEG markers to that end in major depressive disorder (MDD). They showed that TMS-EEG measures are promising markers to predict response to treatment with brain stimulation, in MDD. Further, these measures may help to non-invasively target cortical regions related to MDD (Strafella et al.).

Repetitive TMS can also be applied as a novel intervention for smoking cessation. Shevorykin et al. studied high-frequency rTMS and delay discounting as a new therapeutic target for smoking cessation. Their preliminary findings supported delay discounting as a possible therapeutic target and suggested that an increase in duration and intensity might lead to greater effect sizes in long-term smoking cessation.

Research on motor related effects of TMS have been also matter of debate and improvement recently. TMS elicits motor-evoked potentials (MEPs), measured by electromyography, but non-MEP points may also affect the estimation of the size and centroid of the excitable area. Jin et al. reported in this Research Topic that the incorporation of non-MEP points can improve the estimate of the active area, suggesting TMS approaches that do not consider the non-MEP points are more likely to overestimate the regions of excitability. Examining motor control of other regions e.g., the lumbopelvic musculature is of high interest to those studying low back pain (LBP). This mapping was done by Jordon et al. utilizing TMS and is also reported in this Research Topic.

The investigation on TMS as a novel type of treatment for several clinical conditions is growing fast due to its safety and non-invasive profile. This has been also the case for pain therapy. However, as reported by the work of Li et al., prospective, multi-center, large-sample, randomized controlled trials would be valuable to assess the effectiveness of TMS parameters in pain.

TMS has great potential to study/interfere with cognitive processes and help disentangle their underlying neuronal networks. Luber et al. took great advantage of TMS to interfere with a deception task and provide spatio-temporal information about the neural activity underlying deception action. In this line, Dong et al. stimulated the cerebellar swallowing cortex with high-frequency rTMS to investigate the effects and possible mechanisms of rTMS on swallowing-related neural networks, with resting-state functional magnetic resonance imaging (rs-fMRI).

TMS applications are really expanding in both health and disease. We summarized here incredible findings related to stroke, depression, pain, smoking, or normal brain function mapping. To finish, we highlight the work by Wang et al., showing that rTMS may also improve the visual function in strabismic amblyopic patients. This is an example of the new meaningful applications that are emerging associated with the broader investigation of this promising therapeutic technique.

## Author contributions

All authors listed have made a substantial, direct, and intellectual contribution to the work and approved it for publication.

